# Determinants of dropout from the maternal continuum of care in Ethiopia, multilevel analysis of the 2016 demographic and health survey

**DOI:** 10.1371/journal.pgph.0003641

**Published:** 2024-09-03

**Authors:** Abraham Sahilemichael Kebede, Geremew Werkeshe Wana, Lire Lemma Tirore, Minyahil Tadesse Boltena

**Affiliations:** 1 Arbaminch University, School of Public Health, Arbaminch, Ethiopia; 2 AfriNet Consulting, Addis Ababa, Ethiopia; 3 Department of Public Health, College of Medicine and Health Sciences, Wachemo University, Hossana, Ethiopia; 4 Armauer Hansen Research Institute, Ministry of Health, Addis Ababa, Ethiopia; PLOS: Public Library of Science, UNITED STATES OF AMERICA

## Abstract

Over the past two decades (2000–2020), Ethiopia achieved significant reductions in maternal and neonatal mortality, with a 72% and 44%, respectively. However, low maternal health service utilization and dropout from the maternal continuum of care remain major health system challenges. This study aimed to investigate individual and community-level determinants of dropout from the maternal continuum of care. We used the recent, 2016 Ethiopian Demographic and Health Survey (EDHS) data. In the maternal continuum of care pathway, i) less than four antenatal care (ANC) attendance, ii) subsequent dropout from skilled birth attendance (SBA) after 4 or more ANC, and iii) dropout from postnatal care (PNC) after attendance of facility delivery were the outcomes for dropout. A Multilevel logistic regression analysis of individual and community level factors (e.g., place of residence, geographical regions) were included in the model. The variation in the outcomes were presented by odds ratio (OR), 95% confidence interval and intra-cluster correlation coefficient (ICC). In the maternal continuum of care pathway higher dropouts were observed from SBA to PNC (85%) and from 4+ ANC to SBA (43.4%). Poorest wealth quantile (AOR = 2.31, 95% [CI = 1.69,3.16]), having no health insurance coverage (AOR = 1.44, 95% [CI = 1.01,2.06]), and high community poverty (AOR = 1.28, 95% [CI = 1.01,1.63]) were associated with having < 4 ANC attendance. Perceived distance from health facility as a big problem (AOR = 1.45, [95% = CI, 1.12,1.88), lower community media exposure (AOR = 1.6, 95% [CI = 1.14,2.23]) and rural residency (AOR = 3.03, 95% [CI = 1.75,5.26]) increased the odds of dropout from SBA after 4+ ANC visits. The dropout from the maternal continuum of care was higher in Ethiopia and postnatal care were the most affected maternal care. Oromia and Somali regions were associated with dropouts from all levels of care. Policy strategies should prioritize geopolitical regions with higher dropout levels. In addition to improving access and quality of institutional health services, designing an alternative strategy for targeted outreach for ANC visits and postnatal checkups is recommended.

## Introduction

Globally, between 2000 and 2015, maternal and newborn deaths were reduced by 44% and 47%, respectively. Despite this remarkable achievement, reports in 2017 showed approximately more than 810 women died daily from pregnancy or childbirth-related complications [1]. Failure to attend health services is the major contributor to the preventable yearly toll of half a million maternal, 4 million neonatal, and 6 million child deaths [2–5]. In addition, it contributes to healthcare inefficiency due to the cost incurred by the underutilization of equipment and personnel and delayed diagnosis and treatment [6–8].

To address this significant public health concern, the World Health Organization (WHO) recommends improving the quality of the health service through clinical care interventions and community outreach to deliver services such as continuum of maternal, newborn and child health care (MNCH), family planning and safe abortion [5, 9, 10]. According to WHO, the "Continuum of Care" for MNCH encompasses integrated service delivery for mothers and children ensuring that women have access to quality care throughout pregnancy, childbirth and the postpartum period. This integrated maternal health care proposes that women receive optimal focused antenatal care (ANC) for at least four or more visits occurring between 8 and 12 weeks, 24 and 26 weeks, at 32 weeks, and between 36 and 38 weeks of gestation [11]. These visits should be linked with subsequent deliveries attended by skilled birth attendants and leading to postnatal care (PNC) in a comprehensive and integrated fashion at individual, family, community and health facility levels [5, 12].

Each component of this continuum of care provides a window of opportunity. For example, goal-oriented and focused ANC contacts can be used to provide regular checkups, screen pregnancy and childbirth-related risks, and predict and treat any adverse maternal and neonatal outcomes [12]. Additionally, it can be used to offer interventions addressing common nutritional deficiencies (Iron deficiency anemia, Vitamin A and Iodine deficiencies) among women in the global south [13].

Despite these opportunities, a higher dropout from the continuum of maternal health services is one characteristic of the health care system in sub-Saharan Africa. A study in Tanzania reported a high dropout rate from maternal care, with only 14.5% of women continuing to use a skilled birth delivery after attending ANC [14]. Similarly, in Nigeria, 38% and 51% dropped out from skilled birth attendance and postnatal care after attending the preceding maternal care, respectively [15]. A secondary analysis of 12 sub-Saharan countries reported consistently low service utilization in at least two levels of MNCH care in Mali, Nigeria, DR Congo and Rwanda [16].

Over the last years, Ethiopia has successfully revitalized its national health policy to address universal health access by improving the lack of human resources in health (HRH) and infrastructure [17, 18]. In 2003, the country launched an innovative health extension program (HEP) which trained and deployed over 38,000 health extension workers [19]. Additionally, to improve access to basics and comprehensive emergency obstetrics care (including a caesarian section) at primary health care levels, task shifting of health professionals, for example, accelerated midwifery training and specialized integrated emergency surgery and obstetrics (IESO) was implemented [18]. In addition, there was an aggressive capital investment to improve health coverage to essential services [20].

Despite all these enacted efforts, the maternal mortality ratio(MMR) and neonatal mortality rate (NMR) in Ethiopia remain among the highest in the SSA region [21]. The high maternal mortality can be attributed to the low utilization of maternal health services [2]. According to the 2016 EDHS, the use of maternal healthcare services was low- 4 or more ANC (32%), institutional delivery (26%) and PNC service coverage (17%) [19, 21, 22]. Beyond coverage, the overall quality of ANC service in Ethiopia is low. For example, components such as counseling, education and support, birth preparedness and screening tests were reported as low as 25% [23].

Physical inaccessibility [24], prohibitive costs [12], and sociocultural practices [25, 26] are among the commonly mentioned reasons for the low utilization of maternal services in Ethiopia. In addition, there are disparities in service utilization among regions [27], places of residence (urban, rural) [28], and between different social and economic classes [29, 30]. A critical and comprehensive understanding of the extent and determinants of dropout from the MNCH service could help design a tailored strategy to improve adherence to the maternal continuum of care.

In this study, dropout from maternal Continuum of Care (CoC) is affected by individual, community, and supply-side (health facility) related characteristics. Individual-level factors included personal characteristics of the mother and the household, such as sociodemographic attributes, reproductive history, and factors related to the mother’s autonomy and decision-making power. Additionally, community-level factors encompassing characteristics of the broader environment in which the mother resides, including the socioeconomic status and educational attainment of the community, media exposure at community level, and geographical and administrative contexts were considered. The community-level variables offer better intervention opportunities to practitioners and policy makers [31]. However, previous studies primarily focused on the role of individual-level factors. In this study, our objective is to comprehensively assess the extent of dropout from the maternal continuum of care and determine the factors associated with the dropout when moving from one stage of the continuum to the next.

## Methods and materials

### Study design and source of data

In this study, we used the recent Ethiopian Demographic Health Survey (EDHS) 2016, a nationally representative survey implemented by the central statistical agency (CSA), Ministry of Health (MOH) and the ICF international. The survey collects data on mortality, morbidity, fertility, use of family planning and maternal and child health. The 2007 Ethiopian population and housing census were used as a sampling frame to select enumeration areas (EAs).

**Fig 1:**
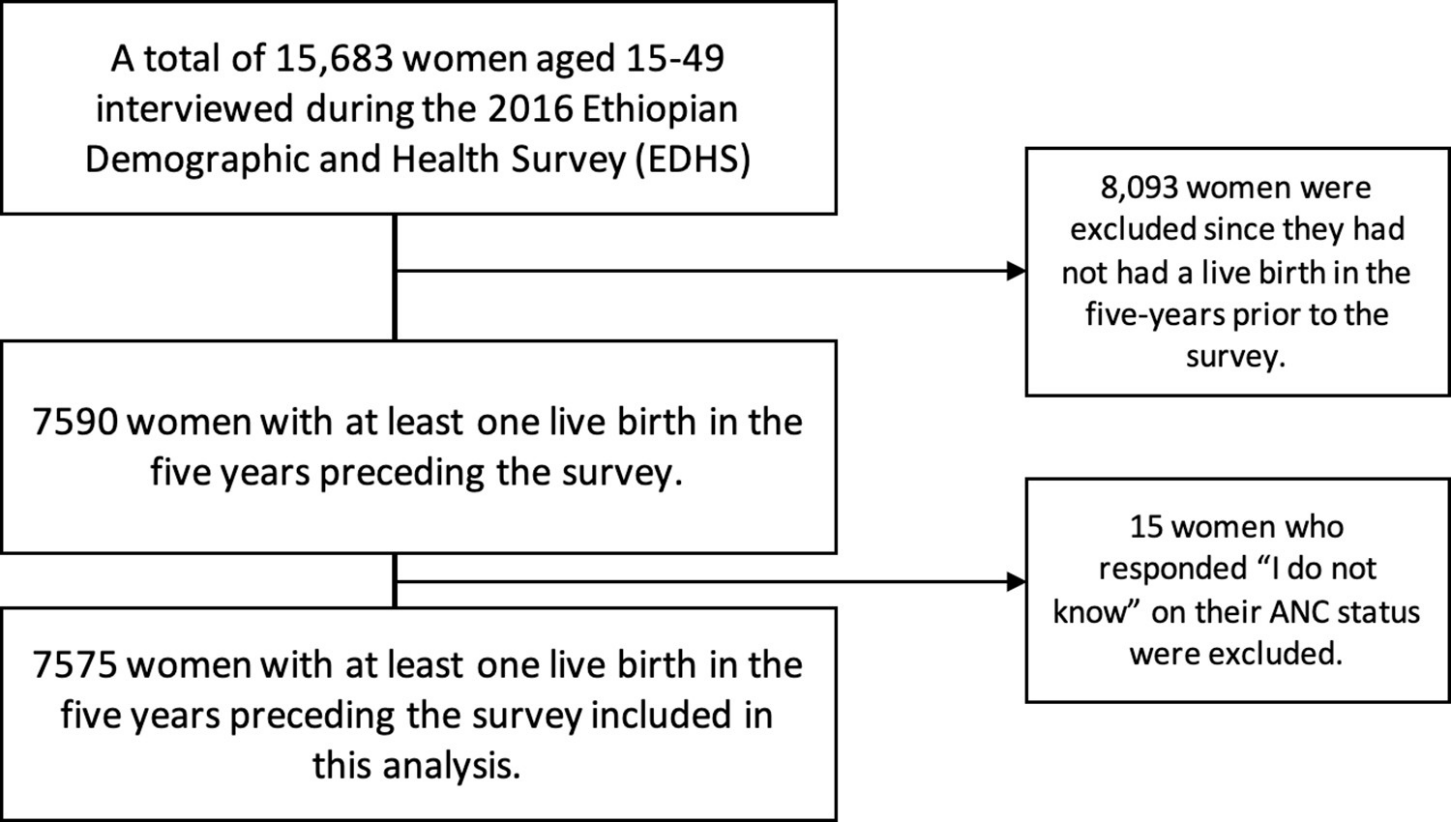
Flow diagram for the sample selection.

The survey employed a two-stage stratified sampling technique. The primary sampling unit constituted regions stratified into urban and rural areas within 21 sampling strata. In the first stage, 645 EAs (202 urban and 443 rural) were selected. In the second stage, 28 households per cluster were selected with equal probability systematic selection from the newly created household listing. Detailed methodology and sampling methods used to collect this data are available elsewhere [21]. A total of 15,683 women aged between 15 and 49 years, either permanent residents of the selected households or visitors who stayed the night before the survey, were eligible to be interviewed. Of this, 8,093 women were excluded since they had not had a live birth in the five years preceding the survey. Out of 7590 women who had at least one live birth in the five years preceding the survey, 15 who responded “I don’t know” were excluded. The final sample size included in this analysis was of 7575 women (See Fig 1).

Since this study was based on secondary data analysis, it does not require ethical approval. However, the Demographic and Health Survey have been approved by ICF institutional IRB and country-specific IRB. The dataset used in study was requested by the first author from the Measure DHS website(www.dhsprogram.com), and permission was granted. Accordingly, the current analysis explores determinants of dropout from the maternal continuum of care pathway (ANC, SBA, and postnatal care) among women who had a live birth in the five years preceding the survey.

### Variable selection and measurement

**Outcome.** The outcome of this study was dropout from the maternal continuum of care. The following three levels of maternal care contact points were used:

- Inadequate ANC: women who did not receive 4 or more focused ANC during their most recent pregnancies were coded “1” and for those who attended ≥4 ANC visits were coded “0”.
- Skilled Birth Attendance (SBA) dropout: women who had 4 or more ANC visits but gave birth with unskilled birth attendant (delivery was not assisted by health professional i.e., doctor, midwife, health officer nurse, and/or health extension worker) were coded as “1” (and otherwise “0”).
- Postnatal care (PNC) dropout: mothers who had 4 or more ANC and sought skilled birth but did not attend postnatal care within the first 2 month of delivery were coded as “1” (otherwise “0”).

#### Independent variables

The independent variables were selected partially based on WHO guidance focusing on the three levels of maternal care utilization: antenatal care, skilled birth attendance and postnatal care. In this model, the dropout from maternal CoC is affected by individual, household, community, and supply-side (health facility) related characteristics [5, 12]. Accordingly, we constructed three groups of independent variables: individual, household, and community-level characteristics.

#### Individual level factors

Maternal age in years, grouped as: below 18, 18–34 and 35 and above. Maternal educational attainment categorized as: no formal education, primary, secondary, and higher education. Mother’s working status, categorized as: currently working or not working; Other pregnancy and reproductive related explanatory variables were number of children ever born to a mother categorized as (one, 2–4 and above 5). Desirability of the pregnancy for the recent birth was asked and the response categorized into wanted then, later, or not at all. The role of partner in the maternal health care utilization (husband’s educational status and occupation status) was also examined. In addition, mother’s autonomy to make decisions to seek for care was included. The “Perceived distance to the health facility”, whether a big problem or not, was considered as a proxy measure to individual’s perceived access to health facilities.

#### Household-level factors

Wealth index is a composite measure of household socioeconomic status, which is based on ownership of selected assets and then analyzed using principal component analysis and classified into five wealth quantiles as: poorest, poorer, middle, richer and richest.

#### Community-level factors

The community-level variables were computed based on the enumeration areas (clusters). Higher and lower community educational status was constructed based on the proportion of mother’s secondary and higher education attendance in the community, using the median value as a cutoff point. Community poverty status used the proportions of mothers from poorest and poorer wealth quantiles in the primary sampling unit, categorized as higher (proportion above the median value) or lower (below the cut off median value). Community media exposure was based on the frequency of listening to the radio, watching TV, and reading newspapers; categorized into higher or lower media exposure based on the median cut off point. The place of residence was categorized into either rural or urban. There are nine administrative regions and two city administrations in Ethiopia. These included Tigray, Afar, Amhara, Oromia, Somali, Benishangul, SNNPR, Gambela, Harari, Addis Ababa and Dire Dawa.

### Data analysis

Data extracted from the DHS program was cleaned and recoded. We used STATA version 14 SE (Stata Corp, College Station, TX) for analysis. Adjustment to account for the complex survey (svyset) method DHS follows was made by weighting the sampling strata, the cluster and sampling weights. The details of this procedure can be found elsewhere [32]. Descriptive statistics that explore the magnitude of maternal health care services in Ethiopia based on different factors were presented using frequency tables and percentages. We run a collinearity diagnostic test to see if the data met the assumption of multicollinearity. Low correlation across the exploratory variables was observed (mean VIF = 1.91).

We performed multilevel logistic regression analysis. The model was fitted to examine the fixed effect (measure of associations) and the random effect (variation component) on the outcome variables; having <4 ANC visits, dropout from SBA after having four or more ANC visits and postnatal care (PNC) after delivering at health institutions. Due to the hierarchical nature of the DHS data, a multilevel analysis was considered appropriate [33].

We conducted a two-level multivariable analysis (individuals nested within clusters) using four models for each outcome variable. The first model was a null model (intercept only), which shows the log-odds of dropping from maternal health services in the absence of covariates. It provides the variance component of random effect to determine whether there is significant variability in dropout from the continuum of care between the communities. The second and third models examined the effects of individual-level and community-level variables on dropout from maternal health care services. The final model included individual (selected independent variables) and community-level variables to predict their respective effect on the dropout from the maternal continuum of care. The magnitude of the association was presented using adjusted odds ratio (AOR) and 95% confidence interval. The variation in the outcome between the clusters (EAs) was measured by the intra-cluster correlation coefficient (ICC). The fitness of the model was tested using the log-likelihood ratio test, which also indicated whether the variance component was statistically significant. In this analysis we had three outcomes with four models each. We presented only the null model and the final model (Model 4) for each outcome. The final model represents the most complete and informative model. The intermediate models (2 and 3) were used to arrive at the final model and contributed into the selection of variables.

The random intercept logit model and the parameters used to estimate the association between explanatory variables and outcome variables were presented in the following model [33]:

$$Log=\beta o+\beta_{1}X_{ij}+\beta_{2}X_{ij\ldots ..}+u_{ij}$$

where π_ij_ is the predicted probability of the outcome variable among dropouts(i) and attended(j) and βo is the constant log odds that y = 1 when the covariates and random effect equals zero (X = 0 and u = 0) which shows the overall intercept in the linear relationship between the log-odds and X. The β_1,2,3_ in the multivariable two-level logistic regression indicates the fixed effects of explanatory variables across all categories and levels i and j (selected sociodemographic variables, socioeconomic, and community level variables). Whereas X_ijk_- is a covariate vector and u_ij—_is the intercept residual (error component) and it is assumed to be normally distributed with variance σ^2^.

The random effect was expressed in terms of intracluster correlation coefficient (ICC) indicating the proportion of total variance on dropout from maternal continuum of care attributable to the community level (clustering) effect.

## Result

**Fig 2:**
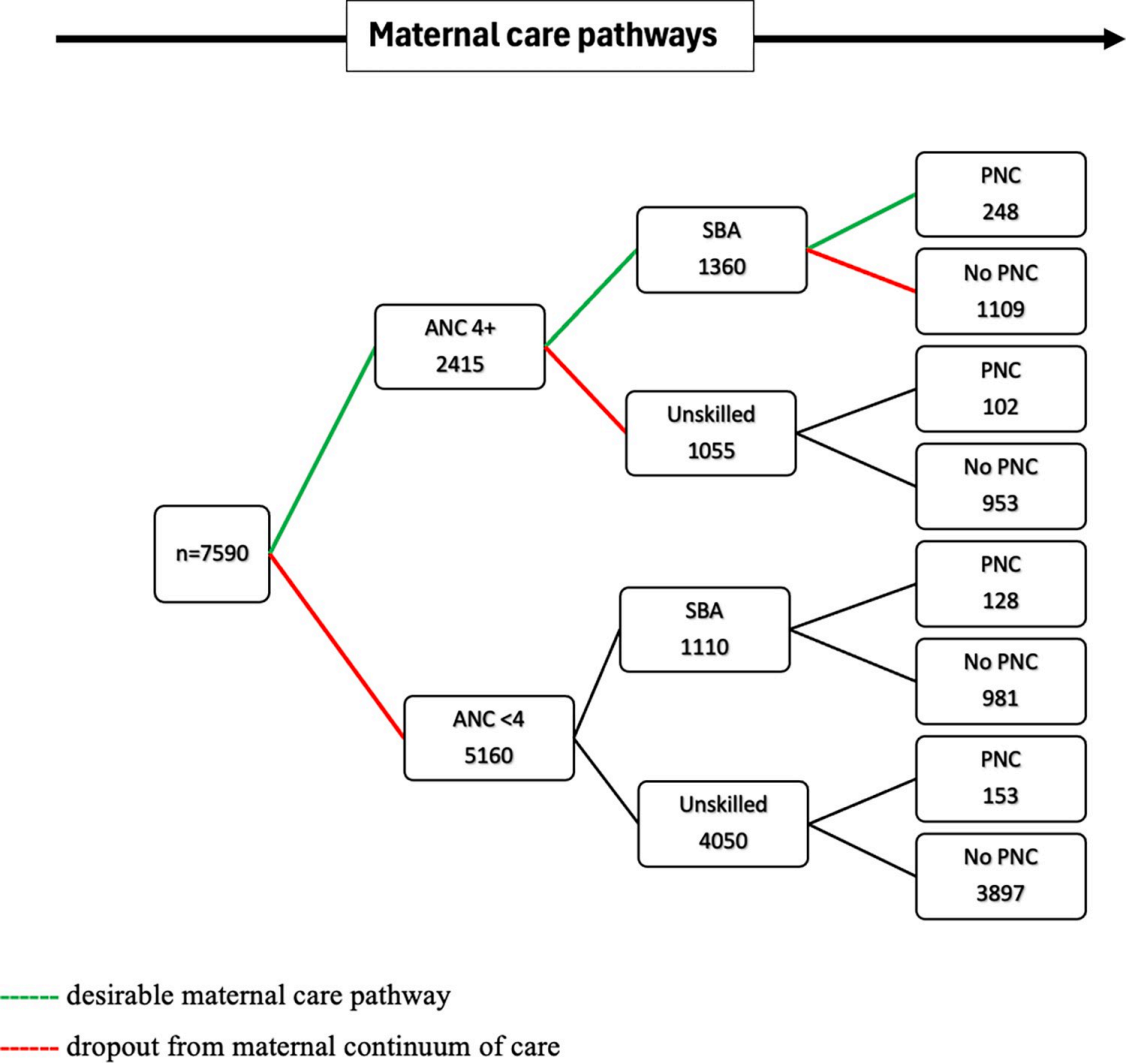
Pathways and patterns of dropout from the maternal continuum of care among women (N = 7575), Ethiopia Demographic Health Survey 2016.

Fig 2 shows the coverage of different levels of maternal service utilization and the pattern of dropout from maternal continuum of care. Out of 7575 mothers who had a live birth five years preceding the survey, 2415 (32%) women attended 4 or more ANC visits, whereas 5160 (68%) of them attended less than four ANC visits. Among those who received 4 or more ANC visits, 56% of them had SBA, whereas 44% dropped out from the SBA care. Only 15.1% attended PNC, and the majority, 85%, dropped out from the PNC service after skilled birth attendance.

Women aged 18–34 years, no formal education, and not currently working had the highest dropout across the maternal CoC. A substantial proportion of mothers without health insurance coverage dropped out from ANC (68%), SBA (44%) and PNC (85%). See Table 1 for the individual-level characteristics of the women included in this study.

**Table 1:** Background characteristics of respondents dropped out from maternal health care services, Ethiopia Demographic and Health Survey, 2016.

Variables	< 4 ANC n = 5160	Had ANC 4+ but no SBA n = 1055	Had ANC 4+, SBA but no PNC n = 1109
	n (%*)	n (%**)	n (%***)
**Age groups (Years)**			
Below 18	174(2.3)	30(1.3)	12(1.1)
18–34	3559(47)	770(31.9)	878(79.1)
Above 35	1428(18.9)	255(10.5)	219(19.8)
**Mother Educational status**			
No Education	3624(75.8)	664(57.4)	413 (37.2)
Primary	1319(61.4)	354(42.7)	391(35.3)
Secondary and above	218(33.6)	37(8.7)	305(27.5)
**Husband Educational status**			
No education	2668(76.9)	451(58.5)	270(26.6)
Primary	1821(66.7)	445(49)	380(37.3)
Secondary and above	440(44.6)	79(14.7)	367(36.1)
**Mother working status**			
Not working	3820(70.6)	803(50.6)	648(58.4)
Working	1341(61.9)	252(30.5)	461(41.6)
**Wealth Index**			
Poorest	1344(81.5)	200(65.7)	91(8.2)
Poorer	1235(74.7)	248(59.3)	151(13.6)
Middle	1138(71.8)	254(56.8)	160(14.4)
Richer	909(63.8)	262(50.7)	205(18.5)
Richest	534(42.3)	91(12.5)	502(45.3)
**Health Insurance**			
No	4986(68.7)	1009(44.4)	1032(93.1)
Yes	175(55)	46(32)	77(6.9)
**Child ever born**			
1	829(57.9)	157(26.1)	353(31.8)
2–4	2097(65.9)	455(41.9)	529(47.7)
5+	2235(78.5)	443(61)	227(20.5)
**Pregnancy wanted**			
Then	379(75.9)	65(54)	45(67.4)
Later	145(75.6)	31(66.1)	14(20.4)
Not at all	59(76.9)	9(52)	8(12.2)
**Own account in a bank or other financial institutions**			
Yes	307(37.2)	81(15.6)	769(69.3)
No	4854(71.9)	974(51.3)	340(30.7)
**Mobile Ownership**			
No	595(43.3)	912(55.7)	616(55.5)
Yes	4566(73.6)	143(18.4)	493(44.5)
**Perceived distance to health facility**			
Big problem	3295(43.5)	623(25.8)	414(37.3)
Not a big problem	1866(24.6)	432(17.9)	695(62.7)

ANC: Antenatal care, PNC: Postnatal care; SBA: skilled birth attendance

*Denominator = the sum of all those who attended and did not attend their ANC

**Denominator = the sum of all those who Had ANC 4 but No SBA and who had ANC and had SBA

***Denominator = sum of all those who had 4 or more ANC, Had delivery care but no PNC within the first 2 months.

Table 2 presents the community-level characteristics of mothers who dropped out from the maternal continuum of care. The majority of the women were rural residents, where 73%, 56% and 87% attended <4 ANC visits, dropped out from SBA and dropped out from PNC service, respectively. Higher dropouts of maternal care (ANC and SBA) were observed among communities with proportionally lower educational levels. Whereas dropout from PNC visits was higher among communities with high community education levels. The dropout was higher across the maternal continuum of care in the Oromia region.

**Table 2:** Community characteristics of women dropped out from maternal service care, Ethiopia Demographic and Health Survey, 2016.

Variables	< 4 ANC n = 5160	Had ANC 4+but no SBA n = 1055	Had ANC 4+, SBA but no PNC n = 1109
	n (%*)	n (%**)	n (%**)
**Place of Residence**			
Urban	356(37%)	49(8%)	437(39.4)
Rural	4804(72.7%)	1006(55.6%)	672(60.6)
**Community Education**			
Low	3652(48.2)	666(27.6)	349(31.5)
High	1508(19.9)	389(16.1)	760(68.5)
**Community poverty status**			
Low	2272(30)	553(22.9)	797(71.9)
High	2888(38.1)	502(20.8)	312(28.1)
**Community media exposure**			
Low	2720(35.9)	568(23.5)	322(29)
High	2440(32.2)	487(20.2)	787(71)
**Region**			
Tigray	228(3)	63(2.6)	187(16.9)
Afar	56(0.74)	8(0.32)	5(0.5)
Amhara	1116(14.7)	247(10.2)	208(18.8)
Oromia	2434(32.1)	374(15.5)	276(24.9)
Somali	236(3.1)	15(0.64)	16(1.4)
Benishangul	47(0.62)	15(0.64)	13(1.2)
SNNPR	986(13)	317(13.1)	251(22.6)
Gambela	12(0.15)	3(0.12)	5(0.5)
Harari	11(0.15)	2(0.06)	5(0.5)
Addis Ababa	21(0.29)	6(0.25)	128(11.6)
Dire Dawa	11(0.15)	6(0.24)	14(1.3)

ANC: Antenatal care, PNC: Postnatal care; SBA: skilled birth attendance

*Denominator = the sum of all those who attended and did not attend their ANC

**Denominator = the sum of all those who Had ANC 4 but No SBA and who had ANC and had SBA

***Denominator = sum of all those who had 4 or more ANC, Had delivery care but no PNC within the first 2 months.

### Determinants of dropout from maternal continuum of care

Table 3 presents the random effect of clusters on <4 ANC attendance, the dropout from SBA and PNC and variations at the cluster level. The variation in the likelihood of attending <4 ANC visits and dropout from SBA services among clusters were significant. The variability between the community in <4 ANC attendance, drop out from SBA and PNC services were 44%, 52% and 19%, respectively.

**Table 3:** Intercept only model and model fitness.

Random effect	< 4 ANC	Had ANC 4+ but no SBA	Had ANC 4+, SBA but no PNC
Community variation	2.59(2.17,3.09) *	3.53(2.71,4.61) *	0.78(0.45, 1.4)
ICC (%)	44%	52%	19.2%
Log likelihood	-4085.3	-1475.5	-789.4

Model: null model (intercept only model) Abbreviations:—ICC: Intra-cluster correlation coefficient, Significance level: *p<0.05

Table 4 presents the multilevel multivariable logistic regression result of selected individual and community level variables on having <4 ANC visits, the dropout from SBA and PNC. The odds of having <4 ANC visits were 1.6 times higher among those with no formal education compared to those with higher education (AOR = 1.6, 95% [CI = 1.21–2.10]). Women from the poorest (AOR = 2.3, 95% [CI = 1.69–3.16]), poorer (AOR = 1.52, 95% [CI = 1.12–3.05]) and middle (AOR = 1.4, 95% [CI = 1.05–1.89]) wealth quantiles were found to have significantly higher odds of having less than 4 ANC visits compared to those from the richest wealth quantiles. The odds of having <4 ANC visits were 1.3 times more likely among communities with higher poverty status compared to those with lower poverty status (AOR = 1.3, 95% [CI = 1.01–1.63]). The odds of <4 ANC attendance was 50% higher among women in communities with low media exposure (AOR = 1.5, 95% [CI = 1.23–1.86]) than women in communities with higher media exposure.

**Table 4:** Multilevel logistic regression analysis of individual and community factors on dropout from maternal continuum of care among women with a live birth in five years preceding 2016 EDHS, Ethiopia.

Variables	< 4 ANC	Had ANC 4 but no SBA	Had ANC 4+, SBA but no PNC
	AOR (95% CI)	AOR (95% CI)	AOR (95% CI)
**Age groups (Years)**			
Below 18	ref	ref	
18–34	1.2(0.79,1.72)	1.0(0.48,1.98)	
Above 35	1.3(0.86,2.03)	0.9(0.41,1.99)	
**Educational status**			
No Education	1.6(1.21,2.10) *	1.8(1.07,2.95) *	2.3(0.16, 34.2)
Primary	1.2(0.92,1.51)	1.6(1.02,2.54) *	4.1(0.42,38.7)
Secondary and above	ref	ref	ref
**Husband’s educational status**			
No education	1.5(1.16,1.82) *	1.7(1.16,2.60) *	0.9(0.61,1.42)
Primary	1.1(0.93,1.41)	1.9(1.32,2.73) *	0.8(0.57,1.12)
Secondary and above	ref	ref	ref
**Mother’s working status**			
Not working	0.98(0.85,1.14)	1.3(1.01,1.71)	1.4(1.15,1.89) *
Working	ref	ref	ref
**Wealth index**			
Poorest	2.3(1.69,3.16) *	1.8(1.02,3.03) *	1.3(0.83,2.13)
Poorer	1.5(1.12,3.05) *	1.2(0.69,1.97)	1.02(0.68,1.53)
Middle	1.4(1.05,1.89) *	1.3(0.76,2.08)	1.2(0.77,1.89)
Richer	1.2(0.86,1.52)	1.2(0.75,1.95)	0.9(0.67,1.45)
Richest	ref	ref	ref
**Health insurance**			
No	1.4(1.01,2.06) *	1.7(0.91,3.06)	
Yes	ref	ref	
**Child ever born**			
1	ref	ref	ref
2–4	1.1(0.89,1.27)	2.1(1.44,2.83) *	1.3(0.98,1.77)
5+	1.1(0.86,1.34)	2.5(1.65,3.74) *	1.1(0.71,1.76)
**Perceived distance to health facility**			
Big problem	1.1(0.97,1.29)	1.5(1.12,1.88) *	1.3(1,1.75)
Not a big problem	ref	ref	ref
**Own account in a bank or other institution**			
Yes	ref	ref	ref
No	1.4(1.23,1.77) *	1.2(0.82,1.85)	1.2(0.87,1.67)
**Mobile ownership**			
No	1.3(1.11,1.64) *	1.6(1.2,2.32) *	1.1(0.77,1.6)
Yes	ref	ref	ref
**Region**			
Tigray	1.5(0.85,22.55)	0.6(0.24,1.56)	1.1(0.62,1.78)
Afar	6.9(3.83,12.3) *	5.6(2.01,15.9) *	1.4(0.64,3.19)
Amhara	4.4(2.56,7.65) *	4.0(1.55,10.2) *	1.3(0.72,2.47)
Oromia	7.3(4.25,12.6) *	3.9(1.55,10.2) *	2.6(1.32,5.04) *
Somali	12.2(6.93,21.6) *	4.2(1.53,11.6) *	4.9(1.93,12.24) *
Benishangul	2.2(1.23,3.81) *	2.4(0.92,6.11)	0.8(0.44,1.56)
SNNPR	2.9(1.70,4.98) *	2.4(0.96,6.0)	1.8(0.98,3.29)
Gambela	4.2(2.38,7.28) *	3.3(1.26,8.54) *	1.3(0.68,2.38)
Harari	7.2(4.14,12.6) *	2.3(0.85,6.14)	9.9(4.14,24.0) *
Dire Dawa	1.3(0.75,2.39)	1.1(0.43,3.02)	3.0(1.55,5.66) *
Addis Ababa	ref	ref	ref
**Place of residence**			
Urban	ref	ref	ref
Rural	1.2(0.86,1.67)	3.1(1.75,5.26) *	1.1(0.67,1.96)
**Community education**			
Low	1.1(0.92,1.41)	1.1(0.81,1.59)	02.4(0.23,24.7)
High	ref	ref	ref
**Community poverty status**			
Low	ref	ref	ref
High	1.3(1.01,1.63) *	1.3(0.87,1.88)	1.1(0.67,1.58)
**Community media exposure**			
Low	1.5(1.23,1.86) *	1.6(1.14,2.23) *	4.8(0.85,27.6)
High	ref	ref	ref
**Random effect**			
Variance	0.5(0.36,0.64) *	0.8(0.47,1.21)	0.4(0.22,0.87) *
ICC (%)	12.6%	18.8%	11.7%
Model fitness			
Log likelihood	-3329	-1073.6	-801.9
AIC	6721.7	2213.1	1977.9

Models with individual and community variables. Abbreviations: -AOR: Adjusted Odds Ratio: adjusted for selected sociodemographic and economic variables and community factors, ICC: Intra-cluster correlation coefficient, AIC: Akaike information criterion; ref: reference group; Significance level:

*p<0.05

Mothers with no formal education were almost two times more likely to dropout from SBA after attending 4 or more ANC visits compared to those who attended higher education (AOR = 1.78, 95% [CI = 1.07–2.95]). Those mothers whose husbands did not attend formal education (AOR = 1.7, 95% [CI = 1.16–2.60) and primary education (AOR = 1.9, 95% [CI = 1.32–2.73]) were more likely to dropout from SBA after four or more ANC attendance. The odds of dropout from SBA maternal care were significantly higher among mothers from the poorest wealth quantiles compared to mothers from the highest wealth quantiles (AOR = 1.8, 95% [CI = 1.02–3.03]). Perceived distance from health facility as big problem increased the likelihood of dropping out from SBA. The odds of dropout from SBA after attending 4 or more ANC visits were 1.4 times more likely among mothers who perceived distance from facility as a big problem compared to those who did not (AOR = 1.5, 95% [CI = 1.12–1.88]). Living in Afar (AOR = 5.6, 95% [CI = 2.01–15.9]) and Somali (AOR = 4.2, 95% [CI 1.53–11.64]) regions were associated with dropping out from SBA. Also, lower community media exposure was associated with higher dropout rate from the SBA (AOR = 1.6, 95% [CI = 1.14–2.23]).

Living in Harari, (AOR = 9.9, 95% [CI 4.14–23.95]), Somali, (AOR = 4.9 95% [CI 1.93–12.3]), Dire Dawa, (AOR = 3, 95% [CI = 1.55–5.66]), and Oromia (AOR = 2.6, 95% [CI = 1.32–5.04]) regions were found to be significantly associated with the dropout rate from PNC visits after SBA.

## Discussion

In this study we investigated, the extent and determinants of dropout from recommended maternal care along the continuum of care from pregnancy to postdelivery in Ethiopia using the EDHS 2016. Higher dropout from the maternal continuum of care was reported compared to studies conducted in other sub-Saharan African countries [14, 15]. On the multilevel logistic regression analysis: literacy level, wealth index, and perceived distance from health facility indicated a significant association with dropout from at least one level of maternal continuum of care. Media exposure at the community level showed a significant association with dropouts from ANC and skilled birth attendance.

Higher and significant disparities among the geopolitical regions in dropout from the maternal continuum of care were observed. Somali and Oromia regions had a significant dropout from all levels of maternal continuum of care. While Afar, Amhara, and Gambella had a significant dropout from at least two levels of maternal continuum of care (ANC and SBA). These regional variations could be attributed to socio-culturally constructed preference for delivering at home by traditional birth attendants. This finding is supported by previous qualitative investigations, which have reported variations across cultures and how cultural contexts shape individual health-seeking behaviors [25, 34]. In-depth research on exemplars and a comparison of high- and low-achieving regions will illuminate the reasons and contextual factors underlying the regional disparities. In addition, designing an alternative strategy such as home-to-home ANC visits and postnatal checkups by mid-level health professionals and integrating the service with other well-adapted services such as immunization might help in averting the dropout and improve service utilization.

Literacy is one of the vital dimensions in human developmental indices. Mothers not having formal education showed association with dropout from ANC and SBA services. This finding was in line with studies conducted in Ethiopia [27, 35], Ghana [36], Cambodia [37] and Nepal [38]. The consistency among these findings reinforces the vital role literacy plays in the overall health-seeking behavior. Primary study conducted in the Northern part of Ethiopia reported higher educational attainment was associated with better awareness of danger signs and subsequent health care attendance [39]. Evidence has also suggested a possible correlation between women empowerment and improved maternal health care service utilization [40]. Empowering women through education, asset ownership and decision-making power significantly reduces both income and multidimensional poverty, especially in rural areas [41]. Consequently, this will have a positive impact on maternal health service utilization.

The role of formal education reducing delay type one, delay in decision to seek care, has been well-established in public health research. Girl child education is one of the most salient interventions, enabling and empowering women to exercise autonomy and control, significantly improving their decision-making abilities in matters related to access and availability of health care services [42, 43]. Efforts to strengthen policies addressing girl child and young women’s education should continue through intersectoral collaborations (education, health and social welfare). Beyond formal education, prioritizing informal learning and support platforms through peer-to-peer learning, positive role models at the community level and using established neighborhood networks such as the Women Development Army (WDA) and community health extension workers is crucial for promoting health-seeking behavior.

Similarly, husband education was also found to be an important determinant of dropout from maternal continuum of care. This finding reiterates the importance of the male involvement in maternal care services and how the husband’s educational status is equally important to reduce the risk of dropout from maternal care service. This finding was similar with a study conducted in Nigeria [44]. Similarly, a systematic review on the effect of male involvement on maternal health care indicated to be an effective strategy to increase healthcare service attendance, family planning uptake and avoid delays [45].

Household wealth index was associated with dropout from ANC and SBA services. This finding was in agreement with different studies done elsewhere in the developing regions [46, 47]. In Ethiopia, the MNCH services are being rendered for free. However, households from the poorest wealth quantile significantly dropout from maternal health care services. This might be attributed to the indirect cost of transportation (tariffs, road qualities, inaccessibility of cheap transport facilities) and health facilities related costs including physical accessibility and availability of health care services for medical complications. A multisectoral collaboration aimed at empowering women in terms of owning financial properties and safety net programs, where women can play a productive role and engage in economic activities should be encouraged.

Perceived distance from the health facility predicted dropout from SBA. This finding was in line with studies from Ethiopia and Nigeria [15, 48]. Physical inaccessibility, either perceived or actual, continues to be a major barrier to the maternal continuum of care. In order to reduce this physical barrier, the government have recently employed a new birth preparedness and readiness strategy, maternity waiting homes, where a mother can stay and receive health care services (ANC and delivery) within the vicinity of the health facility. Despite this intervention, home delivery continues to be high. Further study on supply-side barriers and mothers lived experience of home delivery preference will illuminate why women in Ethiopia continue to deliver at home regardless of receiving optimal ANC visits.

Rural-urban disparities continue to be the source of inequality in maternal health care utilization. This finding highlights the widening divide in access to information, health service availability, literacy, financial ownership, and gender inequality between urban and rural communities. We observed higher odds of dropout from maternal continuum of care among rural residents. Tailored community outreach programs aimed to improve awareness of maternal health services availability focusing on the rural community is recommended. Furthermore, community level media exposure was significantly associated with attending <4 ANC visits and dropping out from SBA. This finding underscored the unprecedented role of media in improving health literacy. Using local communications strategies and working closely with media is imperative in reaching a disadvantaged community.

This study provides a comprehensive assessment of the individual and community level factors determining dropout from maternal care in Ethiopia. However, there are few methodological limitations. One, the nature of the study design fails to establish a temporal relationship. Implying caution should be taken when making inferences regarding causal associations. Second, there might be a possible recall error, as the data were collected from the five years preceding the survey. The distance to the health facility was measured based on the individual’s perceptions, which might not reflect the actual distance from the health facilities. Thus, their perceptions could be confounded by the individual’s context, including favorableness of the road and availability of transport infrastructure.

## Conclusion

Our research highlights the importance of investigating dropouts from maternal continuum of care. The dropout from the maternal continuum of care was higher in Ethiopia compared to other African countries. Postnatal care being the most affected maternal care service. Having no formal education, being from the poorest wealth quantile and low community media exposure were associated with dropout from at least two maternal health care points. Significant disparities among the geopolitical regions in dropout from maternal continuum of care were observed. There were significant dropouts from all levels of maternal care in Oromia and Somali regions. Policy strategies should prioritize geopolitical regions with higher dropout rates and promote the sharing of best practices through platforms connecting high and low achieving regions.

Grassroots-level policies that accelerate girl’s child education and women’s empowerment by enhancing their agency, asset ownership and decision-making power will improve maternal healthcare service utilization. Integrating maternal health services with other well-adapted services, such as immunization, might improve utilization and reduce dropouts from maternal health services. Our study demonstrates the need for targeted interventions to improve adherence to maternal continuum of care completion, including outreach programs, improving access to health facilities, and enhancing the quality of ANC services. A tailored strategy combining social behavioral communication and awareness raising campaigns through established neighborhood networks and positive peer models delivered by community health workers and the women development army is recommended. This requires further contextual and in-depth investigations to understand the specific barriers to and facilitators of maternal healthcare utilization in different communities.
